# A Pragmatic Clinical Trial to Compare the Real-World Effectiveness of V-Go versus Standard Delivery of Insulin in Patients with Advanced Type 2 Diabetes

**DOI:** 10.36469/9731

**Published:** 2019-03-27

**Authors:** Mark J. Cziraky, Scott Abbott, Matt Nguyen, Kay Larholt, Elizabeth Apgar, Thomas Wasser, Poul Strange, Leon Shi, H. Courtenay Harrison, Beverly Everitt, Lynn Nowak

**Affiliations:** 1 HealthCore, Inc., Wilmington, DE; 2 Valeritas, Inc., Bridgewater, NJ; 3 Center for Biomedical Innovation Massachusetts Institute of Technology, Cambridge, MA; 4 Integrated Medical Development, LLC, Princeton Junction, NJ; 5 Endocrinology Consultants, Virginia Beach, VA

**Keywords:** pragmatic clinical trial, type 2 diabetes, insulin delivery device, insulin, v-go

## Abstract

**Background:** Many patients with type 2 diabetes mellitus (T2DM) do not have adequate glycemic control, leading to poor patient outcomes and high healthcare costs.

**Objective:** This prospective pragmatic clinical trial evaluated V-Go, a wearable insulin delivery device, compared with standard treatment optimization (STO) among insulin-treated patients with T2DM in a realworld, community-based practice setting.

**Methods:** Study sites, rather than individual patients, were randomized to V-Go or STO via cluster randomization. Patients were treated according to routine clinical practice and followed up to 4 months. T2DM medications and supplies were purchased utilizing usual insurance and co-pay systems. The primary analysis was an unadjusted treatment group comparison of glycosylated hemoglobinA1c (HbA1c) change from baseline to end of study (EOS). A cost of therapy analysis was completed on patients who had received comparable baseline T2DM treatment with multiple daily basal-bolus insulin injections (MDI).

**Results:** Analysis included 415 patients (169 V-Go, 246 STO) enrolled from 52 US sites. Mean baseline HbA1c (9.6%) was higher in V-Go (9.9%, range 8.0% - 14.2%) than STO (9.3%, range 7.9% - 13.9%, p <.001). HbA1c decreased from baseline to EOS in both V-Go (-1.0%, p<.001) and STO (-0.5%, p<.001); V-Go had significantly larger decrease (p=.002). V-Go had a significant reduction (p<.001) in mean insulin total daily dose (TDD; 0.76 U/kg baseline, 0.57 U/kg EOS), not seen in STO (0.72 U/kg baseline and EOS). The MDI group included 95 (56.2%) V-Go and 113 STO (45.9%) patients. Mean baseline HbA1c was significantly higher in V-Go (9.9%) than STO (9.4%). V-Go also experienced larger decrease in HbA1c from baseline (-1.0%) than STO (-0.36%) (p=.006) with a decrease in TDD, while STO TDD remained unchanged. EOS mean per patient per day cost of diabetes treatment was lower for V-Go ($30.59) vs STO ($32.20) (p=.006). V-Go was more cost effective than STO ($24.02 per 1% drop in HbA1c vs $58.86, respectively).

**Conclusions:** This pragmatic clinical trial demonstrated improved HbA1c levels, lower cost, and decreased insulin dose in patients with T2DM initiating V-Go vs STO in a real-world community-based practice setting. Observed baseline HbAlc indicated use of V-Go in more difficult to manage diabetes patients.

## Background

Type 2 diabetes mellitus (T2DM), affecting approximately 30 million Americans,^1^ is progressive: treatment intensifies as glycemic control declines, eventually leading to subcutaneous insulin therapy.^2^ Despite treatment intensification, at least half of patients using insulin fail to attain targeted glycosylated hemoglobinA1c (HbA1c) levels below 7%.^2–5^

Patient self-reported adherence to insulin regimens ranges from as low as 43% up to 86%, with more flexible regimens and lower patient costs associated with improved adherence.^6^ Insulin therapy adherence decreases with increasingly complex dosing regimens, including numerous daily injections. As T2DM progresses, basal-only insulin regimens become inadequate in controlling glycemic levels, necessitating the addition of bolus insulin regimens typically administered in multiple daily injections (MDI). MDI regimens have specifically been shown to impose an increased burden on both patients and caregivers,^7^ potentially resulting in total daily insulin doses below that which were prescribed due to low treatment adherence.^8^

Poorly controlled T2DM is associated with higher costs, and even small improvements in glycemic control could result in substantial cost savings for third party payers.^9^ Adherence is affected by the insulin delivery device, therefore switching a patient to an easier-to-use device may have a notable effect on treatment adherence.^10–12^ The V-Go insulin delivery device is the first fully disposable, wearable device for the delivery of basal-bolus insulin therapy in adults with T2DM. The device delivers continuous subcutaneous insulin (insulin lispro, rDNA origin or insulin aspart, rDNA origin) in preset basal rates of 20, 30, or 40 units (U) in one 24-hour period, as well as on-demand bolus dosing of up to 36 U in 2 U increments. This delivery method mimics the physiologic distribution of insulin, which may enhance efficacy of insulin therapy.^13^ The V-Go device is discreet, easy to use, and the basal and bolus capability of the device eliminates the need for MDI – all of which may help improve patient compliance with insulin therapy.^13^

The V-Go device has demonstrated efficacy in controlled clinical settings,^14^ and results of small case studies and nonrandomized observational studies have demonstrated effectiveness in everyday usage.^15,16^ Following initiation of insulin treatment with V-Go, significant improvements were observed in glycemic control,^7,8,17–22^ as well as reductions in insulin requirements^8,17,18,20,21^ and lower diabetes-related treatment costs.^7,18,19^ While these effects were observed in a variety of patient populations and settings, all of these studies were retrospective designs and thus were limited in their analysis by reliance on medical records and potential selection bias towards patients with available data. Only two of the studies compared the use of V-Go with a control group.^7,18^ To date, only one study prospectively evaluated V-Go in a real-world practice setting.^23^ Although that study demonstrated reduced HbA1c levels and decreased insulin requirements with use of the V-Go device, it lacked a comparator group.

The current study was designed to fill this gap in evidence. It sought to prospectively assess the effectiveness of the V-Go insulin delivery device compared with standard insulin delivery in adults with T2DM in a real-world, community-based practice setting.

## Methods

### Study Design and Patient Selection

This was a pragmatic clinical trial conducted in adults with T2DM treated with insulin in a real-world, community-based practice setting between September 2013 and April 2016. Physician practice study sites were recruited from clinicians identified as providing care for adult insulin-treated T2DM patients through administrative claims analysis of the HealthCore Integrated Research Database (HIRD^SM^). The HIRD is a large, clinically rich, administrative healthcare database with a broad population base representative of the US population overall.^24^ Study sites (52 total), rather than individual patients, were stratified by four US geographic regions (Northeast, Midwest, South and West) and randomized to either V-Go or standard treatment optimization (STO) via cluster randomization. Study sites enrolled patients in either the V-Go or STO control group depending on the study site randomization assignment. At clinics assigned to V-Go, patients who *a priori* did not want to use V-Go could not participate in the study.

In keeping with the pragmatic design, inclusion and exclusion criteria were minimally restrictive. Eligible patients were offered participation by their physician while discussing the course of routine care. To meet inclusion criteria, patients were required to be between 21 and 80 years old at study enrollment; able to read and understand English; have a physician-confirmed diagnosis of T2DM; have baseline (within 4 weeks) HbA1c 8% to 14%; be currently using insulin therapy with total daily dose of 30 U to 120 U; be willing to go to the physician’s office for follow-up visits and to complete the patient-reported outcome instrument; be willing and able to understand and sign a written informed consent form. Patients were excluded from the study if they had current or planned use of an insulin pump for diabetes management; they were currently participating in any clinical study; they were pregnant, lactating, or intending to become pregnant; were currently using chronic systemic steroids; or they had a diagnosis of type 1 diabetes.

Consistent with the pragmatic design, there were very few study-specific evaluations. Upon enrollment, study physicians treated patients according to routine clinical practice and followed them for a minimum of 3 months, and up to 4 months, to collect study data. Demographic, clinical (height, weight, HbA1c, selected comorbid conditions, concomitant diabetes medications), and insulin regimen data were collected at baseline. Any on-study changes to insulin therapy were recorded as patients presented to the physician as part of standard care. Patients had an end of study (EOS) visit approximately 3 months + 30 days after baseline to collect weight, HbA1c, and insulin regimen data. The EOS had a wide window of up to 30 days after the 3-month time point so as to not impose a schedule on study physicians’ routine clinical practice. Patients completed the Treatment-Related Impact Measures (TRIM^a^)-Diabetes Device, a brief treatment satisfaction questionnaire, at baseline and EOS.^25–27^

Patients enrolled at sites randomized to V-Go were trained on using the device, given starter kits, and discontinued all other insulin therapy. Study staff treated all patients under normal practice conditions, making follow-up phone calls within 3 to 6 days of initiation to make any necessary insulin dosing changes. Patients were treated according to routine clinical practice of their physician, without forced or mandated protocols or titration regimens, at the respective study sites. All treatment medications and supplies were purchased utilizing traditional insurance and co-pay methods as done in usual care models.

The study protocol and all documents were reviewed and approved of by the Quorum Review Institutional Review Board (File #28385) prior to implementation of any study procedures.

### Data Collection

Demographic and clinical data were collected either directly from patients or abstracted from the patients’ medical records and entered into the electronic Case Report Form (eCRF) at study visits (baseline and EOS) by trained study personnel. Patients completed the TRIM-Diabetes Device questionnaire by pen and paper at baseline and EOS, and site study personnel entered the completed forms into the eCRF. Insulin regimen data were collected at baseline and any time there was a change to a patient’s insulin regimen throughout the study.

### Study Outcomes

The primary endpoint was change in HbA1c from baseline to EOS. Baseline HbA1c was defined as the most recent diagnostic HbA1c laboratory value within 4 weeks prior to the baseline visit. EOS HbA1c was defined as that obtained closest to EOS, at least 3 months (+30 days) after the baseline visit. Due to the pragmatic design, in some cases HbA1c tests were performed outside the baseline or EOS visit windows. For these cases, the HbAlc obtained closest to the respective time point was used. The secondary endpoints included patient treatment satisfaction (TRIM-Diabetes Device^25–27^), insulin total daily dose (TDD), and cost of diabetes treatment.

The TRIM-Diabetes Device scale measured the impact of diabetes treatment on a patient’s function and well-being. The TRIM-Diabetes Device consists of 8 items from which two subscales are generated: Device Function (5 items) and Device Bother (3 times). Each item utilizes a 5-point Likert scale and assesses patient satisfaction with their device over the past two weeks. Responses are summed and transformed into a 0-100 scale, where higher scores represent higher device satisfaction.^26–28^ Total and subscale (ie, Device Function and Device Bother) scores were calculated for baseline, EOS, and change from baseline.

TDD (sum of total daily basal and bolus insulin) was calculated in U per day and U per kg body weight for baseline, EOS, and change from baseline. Baseline TDD was calculated from the insulin regimen prior to commencement of any study procedures, ie, prior to initiation of V-Go for patients randomized to the V-Go arm. EOS TDD was the insulin regimen collected closest to EOS. For patients whose insulin regimen did not change during the study, the baseline regimen was used for EOS TDD.

The cost endpoint was the mean per patient per day (PPPD) cost of diabetes treatment, including all concomitant diabetes medications and devices, using Wholesale Acquisition Cost (WAC) at EOS. Cost ($) of diabetes treatment per 1% drop in HbA1c was calculated based on the sample means: WAC costs for all diabetes treatments divided by mean change in HbA1c.

### Statistical Analysis

All analyses were performed using SAS® version 9.4 or higher computer software. The significance level used in all statistical analyses was 5% (two-sided). While physician practices were the unit of randomization for this study, patients were the unit of analysis. Descriptive statistics (mean, median, standard deviation [SD], and range for continuous variables; counts and relative frequencies for categorical variables) were used to summarize patient characteristics, treatments, and outcomes for the study population overall and by treatment group. All change from baseline variables were calculated as absolute change = EOS – baseline. Between-group differences in outcome variables were evaluated using independent t-tests. Within-group differences (ie, from baseline to EOS) were evaluated using paired t-tests.

Statistical analyses were consistent with pragmatic research and used the observations of patient treatments unobstructed by adjustments in the methodology and statistical approach, so as not to interfere with the real-world setting of both treatment patterns practiced by clinicians, as well as the analysis methods determined to be appropriate *a priori*. As a result of these restrictions, all data analysis was unadjusted; observations of recorded testing (HbA1c follow-up measures, for example) were not controlled, but rather reported as collected.

The analysis population was defined as patients who met all inclusion criteria, none of the exclusion criteria, and who had an EOS HbA1c measurement.

Additional analyses were completed on the population with MDIs, a subset of the analysis population that had comparable baseline diabetes treatment with 3 or more daily insulin injections including both basal and bolus insulin injections.

## Results

### Study Population and Baseline Characteristics

A total of 585 patients with T2DM (288 V-Go, 297 STO) were identified from 52 clinical sites (29 V-Go, 23 STO). Of these, 415 patients (70.9%) had a valid post-baseline EOS HbA1c value and were included in the analysis population (169 V-Go, 246 STO; Appendix Table 1). Reasons for not completing the study included declining study participation/refusing V-Go initiation (45 V-Go), early study withdrawal or loss to follow-up (42 V-Go, 50 STO), V-Go related discontinuations (32 V-Go), and death (1 STO). The median follow up time was 97 days for the analysis population overall, and did not differ between V-Go (median 97 days, interquartile range (IQR) 24 days) and STO (median 98 days, IQR 20 days) treatment groups (p=.692).

The majority of patients included in the analysis were white (62.4%) and one quarter of the population was black/African American (25.8%). More women (54%) than men comprised the study population, with a mean age of 59.8 years (range 23 to 80 years; Table 1). There was a wide variation in baseline weight (mean 220.7 lbs, range 111 lbs to 447 lbs) and body mass index (mean 35.3 kg/m^2^, range 17.9 kg/m^2^ to 102.7 kg/m^2^). Baseline HbA1c levels ranged from 7.9% to 14.2% with an overall group mean of 9.6%. The mean TDD at baseline was 71.9 U/day (or 0.74 U/kg) and was similar between treatment groups. Nearly all patients had at least one comorbid condition (96.4%) with hypertension (83.1%) and hyperlipidemia (74.5%) being the most commonly reported.

Baseline clinical and demographic characteristics were largely similar between the V-Go and STO groups. While there were statistical differences in certain characteristics at baseline, many of these statistical differences were not clinically meaningful. Notably, mean baseline HbA1c levels were significantly higher in the V-Go group (9.9%, range 8.0% to 14.2%) compared with the STO group (9.3%, range 7.9% to 13.9%, p<.001).

**Table 1.** Patient Demographic and Baseline Characteristics (Analysis Population)

**Patient Characteristic**	**Overall (N=415)**	**V-Go (n=169)**	**STO (n=246)**	**p-Value^C^**
Age, years, mean (SD)	59.8 (11.1)	57.7 (11.3)	61.2 (10.7)	<.001
Min, Max	23, 80	25, 79	23, 80	
Gender, n (%)				
Female	224 (54.0)	93 (55.0)	131 (53.3)	0.721
Male	191 (46.0)	76 (45.0)	115 (46.7)	
Race, n (%)				
White	259 (62.4)	98 (58.0)	161 (65.4)	0.06
Black/African American	107 (25.8)	54 (32.0)	53 (21.5)	
Asian	9 (2.2)	2 (1.2)	7 (2.8)	
Native American^a^/Native Alaskan	6 (1.4)	4 (2.4)	2 (0.8)	
Other	34 (8.2)	11 (6.5)	23 (9.3)	
Ethnicity, n (%)				
Non-Hispanic or non-Latino	358 (86.3)	147 (87.0)	211 (85.8)	0.725
Hispanic or Latino	57 (13.7)	22 (13.0)	35 (14.2)	
Weight (lbs), mean (SD)	220.7 (54.8)	213.8 (49.7)	225.5 (57.7)	0.028
Min, Max	111, 447	135, 347	111, 447	
BMI (kg/m^2^), mean (SD)	35.3 (9.0)	33.9 (7.5)	36.3 (9.8)	0.005
Min, Max	17.9, 102.7	20.2, 62.3	17.9, 102.7	
Baseline HbA1c (%), mean (SD)	9.6 (1.4)	9.9 (1.4)	9.3 (1.3)	<.001
Min, Max	7.9, 14.2	8.0, 14.2	7.9, 13.9	
Comorbid conditions, n (%)				
Hypertension	345 (83.1)	139 (82.2)	206 (83.7)	0.69
Hyperlipidemia	309 (74.5)	129 (76.3)	180 (73.2)	0.468
Neuropathy	87 (21.0)	34 (20.1)	53 (21.5)	0.726
Coronary artery disease	63 (15.2)	19 (11.2)	44 (17.9)	0.064
Chronic kidney disease	62 (14.9)	16 (9.8)	46 (18.7)	0.01
Retinopathy	36 (8.7)	8 (4.7)	28 (11.4)	0.018
None	15 (3.6)	7 (4.1)	8 (3.3)	0.633
Other	67 (16.1)	15 (8.9)	52 (21.1)	<.001
Baseline insulin regimen, n	412^b^	166^b^	246	
Basal insulin, n (%)	404 (98.1)	164 (98.0)	240 (97.6)	0.373
Bolus insulin, n (%)	295 (71.6)	112 (67.5)	183 (74.4)	0.126
Basal-bolus insulin, n (%)	287 (69.7)	110 (66.3)	177 (72.0)	0.218
Baseline TDD (U/day), mean (SD)	71.9 (27.1)^b^	71.3 (26.1)^b^	72.2 (27.7)	0.797
Min, Max	30, 120	30, 120	30, 120	
Baseline TDD (U/kg), mean (SD)	0.74 (0.29)^b^	0.76 (0.31)^b^	0.72 (0.28)	0.176
Min, Max	0.24, 1.78	0.24, 1.78	0.24, 1.74	

BMI: body mass index; HbA1c: glycated hemoglobin; SD: standard deviation; STO: standard treatment optimized; TDD: total daily dose ^a^ Native American includes American Indians, Native Alaskans, and Native Hawaiians ^b^ Excludes 3 patients in the V-Go group for whom baseline insulin data was unavailable ^c^ p-value based on independent t-test or χ^2^ test to test the difference between treatment groups

### Clinical Outcomes

Although the baseline mean TDD insulin was similar between the treatment groups, the average TDD in the V-Go group decreased significantly from baseline to EOS (mean change -0.2 U/kg, p<.001), while the TDD for the STO group remained unchanged (Table 2).

A significant decrease in mean HbA1c from baseline (9.6%) to EOS (8.9%) was observed in the overall study population (mean change -0.7%, p<.001; Table 2). Patients in the V-Go group experienced a significantly larger decrease than those in the STO group (-1.0% vs -0.5%, respectively, p=.002).

Concomitant diabetes medication use was evaluated for both treatment groups. A higher proportion of patients in the STO group reported using more concomitant diabetes medications in addition to insulin compared with the V-Go group at EOS. Although a high proportion of patients in both groups used 0 to 2 concomitant diabetes medications at EOS (95.2% V-Go and 89.0% STO), a smaller percentage of patients in the V-Go group used 3 or 4 concomitant diabetes medications than those in the STO group (4.8% V-GO and 10.9% STO).

**Table 2.** Changes in Insulin Use and HbA1c, Baseline to End of Study

	**Overall** **(N=415)**	**V-Go** **(n=169)**	**STO** **(n=246)**	**p-Value**
Insulin Dose				
Baseline TDD (U/day), mean (SD)		71.3 (26.1)^a^	72.2 (27.7)	
Min, Max		30, 120	30, 120	
Baseline TDD (U/kg), mean (SD)		0.76 (0.31)^a^	0.72 (0.28)	
Min, Max		0.24, 1.78	0.24, 1.74	
EOS TDD (U/day), mean (SD)		54.0 (13.3)	71.8 (28.5)	
Min, Max		26, 76	26, 155	
EOS TDD (U/kg), mean (SD)		0.57 (0.16)^b^	0.72 (0.28)^c^	
Min, Max		0.20, 0.95	0.23, 1.50	
Change (EOS – baseline) TDD (U/kg), mean (SD)		-0.20 (0.27)^a,b^	0.00 (0.08)^c^	
Min, Max		-0.84, 0.40	-0.56, 0.50	
p-value^d^		<.001	.397	
HbA1c				
Baseline HbA1c (%), mean (SD)	9.6 (1.4)	9.9 (1.4)	9.3 (1.3)	<.001^e^
Min, Max	7.9, 14.2	8.0, 14.2	7.9, 13.9	
EOS HbA1c (%), mean (SD)	8.9 (1.6)	8.9 (1.5)	8.9 (1.6)	.747^e^
Min, Max	5.6, 6.0	6.3, 15.0	5.6, 16.0	
Change (EOS – baseline) HbA1c (%), mean (SD)	-0.7 (1.6)	-1.0 (1.6)	-0.5 (1.6)	.002^e^
Min, Max	-6.8, 5.9	-6.7, 3.0	-6.8, 5.9	
p-value^f^	<.001	<.001	<.001	

EOS: end of study; HbA1c: glycated hemoglobin; SD: standard deviation; STO: standard treatment optimized; TDD: total daily dose ^a^ Excludes 3 patients in the V-Go group for whom baseline insulin data was unavailable ^b^ Excludes 4 patients in the V-Go group for whom EOS weight was unavailable ^c^ Excludes 10 patients in the STO group for whom EOS weight was unavailable ^d^ p-value based on a paired t-test ^e^ p-value is based on an independent t-test to test the difference in change in HbA1c values between treatment groups ^f^ p-value is based on a paired t-test to test the difference in change in HbA1c values from baseline within each treatment group

### Patient-Reported Outcomes

Physicians reported that 93.5% of patients in the V-Go group used the device as directed. Overall, patients rated both the V-Go and standard diabetes delivery devices highly, with mean scores in the 70s and 80s on a 100-point scale, where higher scores indicate higher device satisfaction. Baseline mean TRIM-Diabetes Device scores, which reflect the device used in the 2 weeks prior to study enrollment, were statistically significantly lower in the V-Go group than in the STO group, with between-group mean differences ranging from 5.2 (p=.012, Bother subscale) to 11.6 (p<.001, Function subscale) points in total and subscale scores. However, on average, the mean TRIM-Diabetes Device scores increased from baseline to EOS for the V-Go group, indicating improvement in patient-reported outcomes, but did not appreciably change for the STO group. (Table 3). The largest difference was observed in the Bother subscale, where the V-Go group averaged a 4.8-point increase from baseline (p=.035 for within-V-Go group difference) compared with a 0.4-point decrease for the STO group (p=.043 for between-group difference). The V-Go group also had a statistically significant increase, or improvement, (mean change 3.3, p=.045) in the Total TRIM-Diabetes Device score whereas the STO group had a mean decrease of 0.2 in the score. (Table 3).

**Table 3.** Baseline to EOS at 3 Months Change in TRIM-Diabetes Device Scores

	**Overall** **(N=415)**	**V-Go** **(n=169)**	**STO** **(n=246)**	**p-Value^a^**
TRIM-Diabetes Device Function				
Baseline, n	410	167	243	
Mean (SD)	80.2 (18.3)	73.3 (20.4)	84.9 (15.0)	<.001
Min, Max	20, 100	20, 100	25, 100	
EOS at 3 months, n	392	156	236	
Mean (SD)	80.9 (18.1)	74.9 (20.2)	85.0 (15.3)	
Min, Max	0, 100	0, 100	20, 100	
Change (EOS – Baseline), n	388	154	234	
Mean (SD)	0.9 (17.6)	2.4 (23.3)	0.0 (12.4)	.239
Min, Max	-75, 80	-75, 80	-40, 40	
p-value^b^	.305	.208	.958	
TRIM-Diabetes Device Bother				
Baseline, n	410	167	243	
Mean (SD)	81.6 (21.8)	78.5 (24.8)	83.7 (19.2)	.012
Min, Max	8.3, 100	8.3, 100	25, 100	
EOS at 3 Months, n	392	156	236	
Mean (SD)	82.8 (21.6)	82.4 (22.3)	83.1 (21.2)	
Min, Max	0, 100	0, 100	0, 100	
Change (EOS – Baseline), n	388	154	234	
Mean (SD)	1.6 (22.7)	4.8 (27.8)	-0.4 (18.4)	.043
Min, Max	-100, 91.7	-91.7, 91.7	-100, 66.7	
p-value^b^	.157	.035	.723	
TRIM-Diabetes Device Total				
Baseline, n	410	167	243	
Mean (SD)	80.7 (16.4)	75.2 (18.3)	84.5 (13.7)	<.001
Min, Max	18.8, 100	18.8, 100	43.8, 100	
EOS at 3 Months, n	392	156	236	
Mean (SD)	81.6 (16.3)	77.7 (18.0)	84.3 (14.6)	
Min, Max	6.3, 100	6.3, 100	31.3, 100	
Change (EOS – Baseline), n	388	154	234	
Mean (SD)	1.2 (15.2)	3.3 (20.0)	-0.2 (10.8)	.051
Min, Max	-53.1, 59.4	-53.1, 59.4	-37.5, 43.8	
p-value^b^	.126	.045	.791	

EOS: end of study; SD: standard deviation; STO: standard treatment optimized; TRIM: treatment-related impact measures ^a^ p-value is based on an independent t-test to test the difference in change in TRIM-Diabetes Device scores between treatment groups ^b^ p-value is based on a paired t-test to test the difference in change in TRIM-Diabetes Device scores from baseline within each treatment group

### Multiple Daily Insulin Injection Subset

Cost and effectiveness analyses were completed on patients (95 V-Go, 113 STO) who had comparable baseline diabetes treatment with 3 or more injections per day including both basal and bolus insulin injections. In this MDI group, comprising 50% of the overall study group, patients in the V-Go group had a higher baseline HbA1c (9.9%) than the STO group (9.4%). The V-Go group experienced a significantly larger decrease in HbA1c from baseline (-1.0%) than the STO group (-0.4%; p=.006). This decrease was observed with a reduction in mean TDD administered to patients in the V-Go group from baseline (75 U/day) to EOS (55 U/day). In contrast, the mean TDD of the STO group remained unchanged from baseline to EOS (77 U/day). Additionally, the mean PPPD cost of diabetes treatment (including all concomitant diabetes medications and devices), calculated using WAC, was lower for the V-Go ($30.59) compared with the STO ($32.20) group. Improved cost effectiveness was seen in V-Go-treated patients, with WAC costs of $24.02 per 1% drop in HbA1c, compared with $58.86 for patients in the STO group (Table 4).

**Table 4.** Glycemic Control, Insulin Dose, and Cost in the MDI Subset

	**V-Go** **(n=95)**	**STO** **(n=113)**	**p-Value**
Baseline HbA1c, %			
Mean (SD)	9.9 (1.5)	9.4 (1.3)	.005
Change (EOS – baseline) HbA1c, %			
Mean (SD)	-1.0 (1.6)	-0.36 (1.5)	.006
Baseline TDD (U/day)			
Mean (SD)	75 (23.1)	77 (27.3)	.624
EOS TDD (U/day)			
Mean (SD)	55 (14.0)	77 (28.2)	< .001
EOS Diabetes treatment cost PPPD, $			
Mean (SD)	30.59 (17.6)	32.20 (32.4)	.006
EOS Diabetes treatment cost effectiveness, $ cost per 1% HbA1c drop^a^			
Mean	24.02	58.86	

EOS: end of study; HbA1c: glycated hemoglobin; MDI: multiple daily insulin injection; PPPD: per patient per day; SD: standard deviation; STO: standard treatment optimized; TDD: total daily dose; ^a^ Cost ($) of diabetes treatment per 1% drop in HbA1c was calculated based on the sample means: WAC costs for all diabetes treatments divided by mean change in HbA1c. Therefore, SD and p-Value were not calculated.

## Discussion

This prospective, pragmatic clinical trial provides real-world evidence of increased glycemic control and patient treatment satisfaction in adults with T2DM initiating treatment with the V-Go insulin delivery device, compared with patients using standard delivery of insulin and treatment optimization. Additionally, patients in the V-Go group had decreased insulin requirements and costs compared with the standard therapy group. This is the first pragmatic trial to specifically study the impact of the V-Go insulin delivery device compared to standard treatment delivery.

On average, the V-Go and STO groups both demonstrated improved glycemic control as evidenced by significant decreases in HbA1c levels in the study population overall as well as by treatment group within the 4-month treatment period. A larger decrease in HbA1c was observed in the V-Go group than in the STO group, suggesting additional benefit beyond routine follow-up. However, the mean baseline HbA1c levels were higher in the V-Go group than in the STO group, suggesting greater diabetes disease severity in patients in the V-Go group, which may have contributed to observed differences in study outcomes between treatment groups. Previous research suggests the greatest gains in glycemic control upon switching to V-Go are seen in patients with higher prior HbA1c levels.^8^ Additionally, differences in the distribution of comorbid conditions between V-Go and STO groups may have contributed to observed differences in study outcomes.

Overall, the study population had a mean baseline HbA1c level of 9.6%. While high, it is also indicative of the real-world nature of this pragmatic clinical study and highlights the unmet need for improved therapy for this population of adults with T2DM. The elevated HbA1c levels are also consistent with previous retrospective studies targeting sub-optimally controlled diabetes, in which mean baseline HbA1c levels ranged from 8.9% to 9.6%.^7,8,18,19^ The mean reduction in HbA1c levels (-1.0%) in the V-Go group was within the range of decreases reported in prior studies of patients initiating V-Go after similar follow-up periods (range -0.8% to -2.0%).^17–20,22^ These prior studies were retrospective and relied on HbA1c data from electronic medical records rather than collected prospectively as in the current study. The V-Go population in the current study also included patients with poorly controlled diabetes (ie, baseline HbA1c levels above 9.5%), which may be more difficult to treat than well-controlled diabetes. While our findings may thus be generalizable to patients with more advanced disease, they may also be generally applicable to the overall population with diabetes given the recent evidence of the population captured in the HIRD.^5,9,29^ In a population of commercially insured patients with diabetes, the mean HbA1c level among patients taking insulin was higher than 8.0%, and approximately 80% of the population had HbA1c levels higher than recommended target level of 7.0%.^5^

Although patients in the V-Go group experienced significant decreases in their insulin requirements, these were accompanied by significant reductions in HbA1c values from baseline to EOS. Patients in the V-Go group reported a 24% reduction in TDD U/day and a 25% reduction in TDD U/kg while patients in the STO group reported no changes in TDD. Our observation of decreased insulin utilization after initiation of V-Go is consistent with previous research^8,18–20,22^ and supports the theory that continuous insulin infusion may be more efficient than insulin delivery via single or multiple injections.^14,18^

Patients in the V-Go as well as the STO group were generally satisfied with their insulin delivery device at baseline and EOS, with average TRIM-Diabetes Device scores in the 70s and 80s on a 100-point scale. Patient satisfaction scores at baseline, ie, satisfaction associated with their insulin delivery device used prior study enrollment, was on average lower in V-Go group compared with STO. However, on average, patient satisfaction scores in the V-Go group increased from baseline to EOS while scores in the STO group remained unchanged. The largest improvement in V-Go scores was in the Bother subscale (mean change of 4.8 points), which represented both a significant change from baseline and a significant difference from the change in the same subscale in the STO group (mean change -0.4 points from baseline to EOS). This finding is consistent with prior research indicating positive patient perceptions of V-Go,^16,21^ but it is the first time the effect has been observed in a large prospective study and in comparison with standard insulin delivery. While this observation is suggestive of increased patient satisfaction with V-Go over other insulin delivery devices, this interpretation is moderated by the observed baseline imbalance in TRIM-Diabetes Device Scores between treatment groups. Although increases in V-Go patient satisfaction measures were small and with uncertain clinical relevance, our results support the theory that patients are less “bothered” by a wearable device such as V-Go, compared to their previous insulin delivery devices, which could lead to improved treatment adherence and ultimately better glycemic control.

The observation of improved glycemic control, coupled with decreased insulin requirements in patients initiating treatment with V-Go compared with STO, persisted in an analysis of the MDI subset. The MDI subset had comparable baseline diabetes treatment, however, as in the overall cohort, mean baseline HbA1c levels were higher in the V-Go group than in the STO group and may have contributed to the observed differences in study outcomes between treatment groups. Similar to the analysis of the overall study population, both V-Go and STO groups in the MDI subset experienced decreases in HbA1c from baseline to EOS. The decrease in the V-Go group was significantly larger than in the STO group (-1.0% V-Go vs -0.4% STO; p=.006) and was similar to the overall V-Go population (-1.0%). In the MDI subset, the mean TDD of the V-Go group decreased significantly from baseline to EOS while it remained unchanged in the STO group. Additionally, the calculated cost of diabetes treatment per 1% improvement in HbA1c was 59% lower in V-Go compared with STO in the MDI subset.

### Limitations

While this pragmatic clinical trial had several strengths, including increased generalizability of the results across ethnicities and races due to the size and composition of the study population, limitations should be noted. There were observed differences in baseline characteristics between treatment groups. However, because these differences were reflective of the pragmatic nature of this study, no statistical controls were put into place to adjust or artificially equate these samples. Some may argue that data analysis adjusted for baseline variation, rather than unadjusted data, would be conducted and reported. However, given the pragmatic design and the real-world context, the unadjusted data analysis following the *a priori* data analysis plan is methodologically appropriate.

In particular, differences indicated greater diabetes disease severity in patients in the V-Go treatment group, possibly due to a bias of more advanced diabetes and perhaps less adherent patients initiated on V-Go, given the cluster randomization scheme. Therefore, the contribution of diabetes disease severity to observed treatment group differences must be considered. Additionally, a higher percentage of patients in the V-Go group did not complete the study compared to patients in the STO group, which may have in part been due to greater flexibility in STO treatment or personal disinclination towards a wearable device. The study objective was to compare insulin delivery via V-Go versus standard of care, as determined by the treating physician according to routine clinical practice. As such, patients enrolled in the STO group were allowed to add or change therapy during the study as consistent with their clinician’s standard practice, whereas patients initiating V-Go must have initiated and maintained V-Go for the study duration. This design constraint may have introduced a differential adherence allowance between treatment groups, which could have contributed to the observed difference in study outcomes.

The pragmatic design had few study-specific evaluations, but simply being followed for study participation may have influenced treatment outcomes. However, study-specific evaluations (other than V-Go-specific instructions) were the same between treatment groups. While both treatment groups had significant improvements in overall HbA1c levels, the decrease in HbA1c levels in the V-Go group was significantly greater than that in the STO group, indicating the device was likely a factor in the result. The follow-up period was relatively short (up to 4 months) and maximal effectiveness of the intervention was likely not attained. The significantly higher baseline HbA1c in the V-Go group compared with the STO group plus previous research suggest the greatest gains in glycemic control after V-Go initiation are observed in patients with higher initial HbA1c levels.^8^ Research also suggests glycemic control continues to improve with longer follow up after V-Go initiation.^8,18,23^

## Conclusions

This is the first large-scale pragmatic study to evaluate the V-Go device in a real-world community-based setting. The population was largely similar to the general diabetes population receiving insulin, with high baseline HbA1c levels and other comorbidities as demonstrated in large epidemiological studies.^5,9,29^ This study provides evidence of increased glycemic control and patient satisfaction coupled with decreased total insulin requirements and diabetes costs in patients with T2DM initiating V-Go for insulin delivery compared with patients continuing standard insulin delivery. Observed treatment group characteristics indicated use of V-Go in more difficult to manage diabetes patients. These findings indicate V-Go may contribute to improved patient outcomes in the population of insulin-treated T2DM patients, and decreased diabetes treatment costs among patients receiving MDI. Real-world studies such as this help define the use of V-Go for insulin delivery as actually used by physicians in practice. Additional research will further examine the real-world populations of T2DM patients that will benefit most from the V-Go delivery device.

## Conflict of Interest

Funding for this study was provided by Valeritas, Inc. Mark Cziraky, Thomas Wasser, and Elizabeth Apgar are employees of HealthCore, Inc., which received funding from Valeritas, Inc. for the conduct of the study. Lynn Nowak was an employee of HealthCore, Inc. at the time of the study. Scott Abbott and Matt Nguyen are employees of Valeritas, Inc. Study design, data collection and interpretation, manuscript drafting and review, and decision to publish were performed solely by the authors.

## Notes

TRIM-D Device^©^ Novo Nordisk, August 2008; TRIM-D Device contact information and permission to use: MAPI Research Trust, Lyon, France.

E-mail: PROinformation@mapi-trust.org – Internet: www.mapi-trust.org

## Figures and Tables

**Figure:** Supplementary Content
